# ﻿A revision of the North American genus *Proctorus* (Coleoptera, Curculionidae, Ellescini) with descriptions of two new species

**DOI:** 10.3897/zookeys.1131.90392

**Published:** 2022-11-23

**Authors:** Jake H. Lewis, Robert S. Anderson

**Affiliations:** 1 Environmental Science Section, Okinawa Institute of Science and Technology, 1919-1 Tancha, Onna-son, Kunigami-gun, Okinawa, 904-0495 Japan Environmental Science Section, Okinawa Institute of Science and Technology Tancha Japan; 2 Department of Natural History, New Brunswick Museum, 277 Douglas Avenue, Saint John, New Brunswick, E2K 1E5 Canada Department of Natural History, New Brunswick Museum Saint John Canada; 3 Beaty Centre for Species Discovery, Canadian Museum of Nature, 1740 Chemin Pink, Gatineau, Quebec, J9J 3N7 Canada Beaty Centre for Species Discovery, Canadian Museum of Nature Quebec Canada

**Keywords:** Museology, new species, rare species, species discovery, taxonomy, weevil, willows

## Abstract

The rarely collected North American endemic genus *Proctorus* (Coleoptera, Curculionidae, Ellescini) has hitherto contained two described species, *P.armatus* LeConte, 1876 and *P.decipiens* (LeConte, 1876). Here, *Proctorus* is revised and two new species, namely *P.emarginatus***sp. nov.** and *P.truncatus***sp. nov.**, are described. Lectotypes for *P.armatus* and *P.decipiens* are designated from known syntypes. All four species in the genus are associated with Salicaceae, but, in addition to differences in external and genital morphology, there is also evidence of differing host plant usage between the species. A photographic key to the four species is provided to facilitate identification.

## ﻿Introduction

The genus *Proctorus* LeConte, 1876 (Coleoptera, Curculionidae, Ellescini) has hitherto contained two described species, namely *P.armatus* LeConte, 1876 and *P.decipiens* (LeConte, 1876), which feed on species in the family Salicaceae and are endemic to North America (O’Brien and Wibmer 1982; Anderson 2002; Bousquet et al. 2013). Members of the genus are rarely collected, and the males possess remarkable modifications (e.g., ventral projections, carinae) on the apical abdominal ventrites (LeConte 1876, 1878). In addition to *Proctorus*, the tribe Ellescini contains several other northern genera that also feed on Salicaceae, namely *Dorytomus* Germar, 1817, *Ellescus* Dejean, 1821, and *Rodotymus* Zumpt, 1932 (Alonso-Zarazaga and Lyal 1999; Anderson 2002; Caldara et al. 2014). The genera *Dorytomus* (100+ species) and *Ellescus* (7 species) are Holarctic in distribution and *Rodotymus* (monotypic) occurs in Kazakhstan and its bordering countries (Alonso-Zarazaga and Lyal 1999; Anderson 2002). Anderson (2002) separated *Proctorus* from the related genera *Dorytomus* and *Ellescus* on the basis of the former possessing basal teeth on the tarsal claws and femoral teeth (*Dorytomus*: simple tarsal claws, with femoral tooth; *Ellescus*: basal teeth on tarsal claws, without femoral tooth). *Proctorus* is currently placed in Ellescina with *Ellescus* (Alonso-Zarazaga and Lyal 1999; Anderson 2002; Bousquet et al. 2013; Caldara et al. 2014; Alonso-Zarazaga et al. 2017) based on shared toothed tarsal claws; the claws are simple in *Dorytomus*, which is generally placed in Dorytomina.

The purpose of this paper is to revise *Proctorus* and describe two new species, *P.emarginatus* sp. nov. and *P.truncatus* sp. nov., from North America.

## ﻿Materials and methods

Specimens were borrowed from public and private insect collections as well as collected in the field. Institution names and associated acronyms used in this work are presented below:

**AFCF**Atlantic Forestry Centre, Canadian Forest Service, Natural Resources Canada, Fredericton, New Brunswick, Canada;

**CAS** California Academy of Sciences, San Francisco, California, U.S.A.;

**CBG** Center for Biodiversity Genomics, Guelph, Ontario, Canada;

**CCCH** Claude Chantal Insect Collection, Varennes, Quebec, Canada;

**CMNC** Canadian Museum of Nature, Ottawa, Ontario, Canada;

**CNCI**Canadian National Collection of Insects, Arachnids, and Nematodes, Ottawa, Ontario, Canada;

**MCZC** Museum of Comparative Zoology, Harvard University, Massachusetts, USA;

**NBM**New Brunswick Museum Insect Collection, Saint John, New Brunswick, Canada;

**PdTC** Pierre de Tonnancour Collection, Terrasse-Vaudreuil, Quebec, Canada;

**RBCM**Royal British Columbia Museum, Victoria, British Columbia, Canada;

**RWC** Reginald Webster Collection, Charters Settlement, New Brunswick, Canada;

**UAMIC** University of Alaska Museum Insect Collection, Fairbanks, Alaska, USA;

**USNM**United States National Museum, Washington, District of Columbia, USA.

Specimens were dissected using standard protocols and genitalia were cleared in a water + KOH solution. The sexes were associated primarily by collection event (i.e., same day and locality). All examined specimens have Unique Specimen Identifier (USI) labels attached that read in the form: JHLRSA_PROC_###. All images were taken using a Leica Z16 APOA camera and LAS images stacking software (Leica Microsystems, Wetzlar, Germany).

## ﻿Results

A taxonomic investigation of the genus *Proctorus* revealed the presence of an additional two species, namely *P.emarginatus* sp. nov. and *P.truncatus* sp. nov., with distinct external and genitalic (male) morphology. The fifth ventrite of males bear modifications (e.g., projections, carinas) that not only define the lineage more broadly, but also contain useful phylogenetic information and allow for unambiguous separation of males of the now four known species. Furthermore, there is some evidence of differing host plant usage between the species (see *P.armatus* species profile). Although females were dissected, no consistent differences in female genitalia were observed and thus females were largely identified by association with males taken during the same collection event (i.e., locality and date). *Proctorusemarginatus* is described here from three male specimens; the female remains unknown. This distinctive species is very rare, apparently restricted to northwestern North America, and has not been collected since 1988, when it was collected in the Northwest Territories. A photographic key to *Proctorus* along with profiles for each species are presented below.

### ﻿Taxonomy

#### ﻿Genus Proctorus

Taxon classificationAnimaliaColeopteraCurculionidae

LeConte, 1876

1C4C54B0-6DCF-528A-91C4-B0C0DEADF5FE

Proctorus LeConte, 1876: 212. LeConte 1878: 620. O’Brien and Wibmer 1982: 94. Alonso-Zarazaga and Lyal 1999: 78. Bousquet et al. 2013: 326.
Encalus LeConte, 1876: 213. Type species: Encalusdecipiens LeConte, 1876 (monotypy).

##### Type species.

*Proctorusarmatus* LeConte, 1876, by monotypy.

##### Gender.

Masculine.

##### Diagnosis.

Length 2.9–4.1 mm. Small, rounded, cuticle dark (black) or dark red and some species with dull orange, transverse stripe on elytra. Cuticle with coarse, white and/or yellow hair-like or more broad scales. Rostrum stout, roughly equal in length to pronotum, and often covered in scales up to antennal insertion. Eyes small and circular to oval, extending somewhat onto the rostrum medially. Antennae reddish with small, oval club. Pronotum as wide as long, coarsely punctate, scaled, and with or without prominent smooth, longitudinal midline. Scutellum not covered densely with bright white scales. Elytra oval in dorsal view, striae with large, deep punctures each bearing a scale. Punctures of elytral striae distinctly larger than those of pronotal disk. Interstrial regions of elytra with 2–4 irregular rows of scales. Fifth ventrite of male modified, with various projections and carinae. Fifth ventrite of female unmodified. Legs with femora toothed. Tarsal claws bearing basal tooth. Aedeagus rounded, subquadrate or emarginate at apex. Internal sac with hook-like sclerite.

###### Species profiles

#### ﻿ Proctorus armatus

Taxon classificationAnimaliaColeopteraCurculionidae

LeConte, 1876

D77BDC11-53AE-5317-8D37-CDB71C088EBF

**Figure 1.**
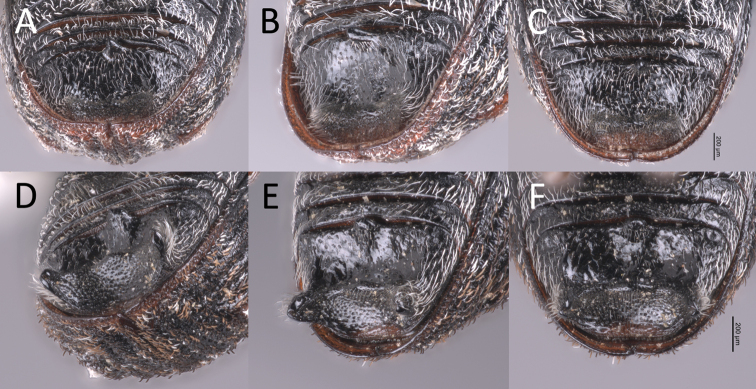
**A–C** male *Proctorustruncatus* fifth ventrite **A** slightly oblique view **B** oblique view **C** ventral (straight-on) view **D–F** male *Proctorusarmatus* fifth ventrite **D** slightly oblique view **E** oblique view **F** ventral (straight-on) view.

Figs 1D–F , 4 , 7C, D , 8A

Proctorus armatus LeConte, 1876: 212 [type locality: south side of Lake Superior (USA)]. LeConte 1878: 620. O’Brien and Wibmer 1982: 94. Alonso-Zarazaga and Lyal 1999: 78. Bousquet et al. 2013: 326.

##### Material examined.

***Lectotype* (here designated): USA**: south side of Lake Superior, Type 5244 (1 female, MCZC), MCZ-ENT00005224.

***Paralectotype***. **USA**: south side of Lake Superior, Type 2 5244 (1 female, MCZC), MCZ-ENT00529966.

##### Non-type material.

**Canada: Alberta**: Tp. 39, Rge. 27, 1 April 1985, B.F. & J.L. Carr, on *Populus* (3, CNCI; 1 CMNC), JHLRSA_PROC_313 – JHLRSA_PROC_315, JHLRSA_PROC_321; Calgary, 4–5 July 1974, C.V. Nidek (1, CMNC), JHLRSA_PROC_322; **Manitoba**: Aweme, 7 April 1925, N. Criddle (1, CNCI), JHLRSA_PROC_317; Riding Mountain Park, 9 June 1937, W.J. Brown (1, CNCI), JHLRSA_PROC_319; Winnipeg, 15 April 1916, L.H. Roberts (1, MCZC), MCZ-ENT00726923; **Northwest Territories**: Fort Smith, 13 June 1988, B.F. & J.L. Carr (1, CNCI), JHLRSA_PROC_320; **Ontario**: Moose Factory, 26 June 1948, W.Y. Watson (1, CNCI), JHLRSA_PROC_316. **USA: Alaska**: Willow, Fishhook Road near Deception Creek (61.7622°N, 150.4603°W), 11 August 2014, R. Progar & S. Bresney, on *Populus* (1, UAMIC) , UAM100378225; Two Rivers (64.88925°N, 147.0871°W), 24 May 2015, S. Melerotto (1, UAMIC), UAM100430466; **Michigan**: Marquette, (2, MCZC (LeConte and Horn Collection)), MCZ-ENT00772797, MCZ-ENT00772798; Marquette, Hubbard & Schwarz (1, USNM), JHLRSA_PROC_330; **New Mexico**: Cloudcroft, 20 July 1978, J.M. Campbell (1, CNCI), JHLRSA_PROC_318.

##### Diagnosis.

Length 3.8–4.1 mm. Body (especially rostrum and femora) dark, although elytra often with orange stripe extending posteriorly from humerus. Protibiae of male dentate on inner edge. Elytra without clear, distinct x-pattern of white scales. Fifth ventrite of male with two prominent ventral projections apico-laterally which are connected by a transverse ridge; also with a single, smaller ventral projection positioned baso-medially. Apical tooth of metatibiae of male straight. Aedeagus in dorsal view slightly rounded to truncate, not emarginate or significantly expanded laterally.

##### Notes on types.

This species was described based on three specimens collected along the south side of Lake Superior. Two examined syntypes in the MCZC bear several types of labels. Both specimens bear a rectangular brown label reading “J.L. LeConte Coll.”, a square red type label reading “Type 5224” and “Type 2 5224”, and MCZC unique identifier labels (see specimens examined). One specimen also bears a rectangular brown label reading “Proctorusarmatus Lec.” and a rectangular white label reading “Jan.-Jul. 2005 MCZ Image Database”. The location of the third (male) syntype specimen is unknown; however, the identity (i.e., *P.armatus*) of that specimen is clear, based on LeConte’s original description. Here, we designate one of the known *P.armatus* syntypes (MCZ-ENT00005224) as a lectotype to fix the identity of this species.

##### Taxonomic comments.

See same section for *Proctorustruncatus*.

##### Remarks.

This species is likely most closely related to *P.truncatus* as males of both species bear a basomedial projection on the fifth sternite (lacking in other species; males only), have a straight metatibial spur (lacking in *P.decipiens*; only males), and are both large and dark in general form. Furthermore, examined specimens of *P.truncatus* and *P.armatus* were only collected from *Populus* when such data was recorded, whereas examined specimens of *P.decipiens* and *P.emarginatus* have only been collected from *Salix* species. Although more field data should be amassed to support this difference in host plant preference, an emerging pattern of differing host plant preference is apparent and supports the hypothesis that *P.truncatus* and *P.armatus* are closely related.

LeConte (1876) remarked that *P.armatus* lacks femoral teeth. However, we note that the four species treated here all possess a distinct tooth on all femora.

#### ﻿ Proctorus decipiens

Taxon classificationAnimaliaColeopteraCurculionidae

(LeConte, 1876)

6217778A-14C3-5B84-8F04-83BD60954A70

**Figure 2.**
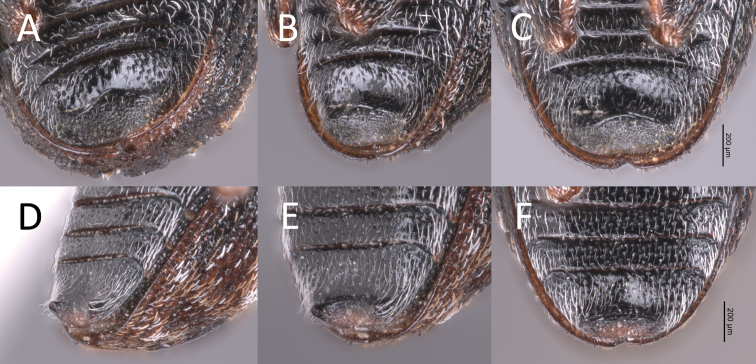
**A–C** male *Proctorusemarginatus* fifth ventrite **A** slightly oblique view **B** oblique view **C** ventral (straight-on) view **D–F** male *Proctorusdecipiens* fifth ventrite **D** slightly oblique view **E** oblique view **F** ventral (straight-on) view.

**Figure 3.**
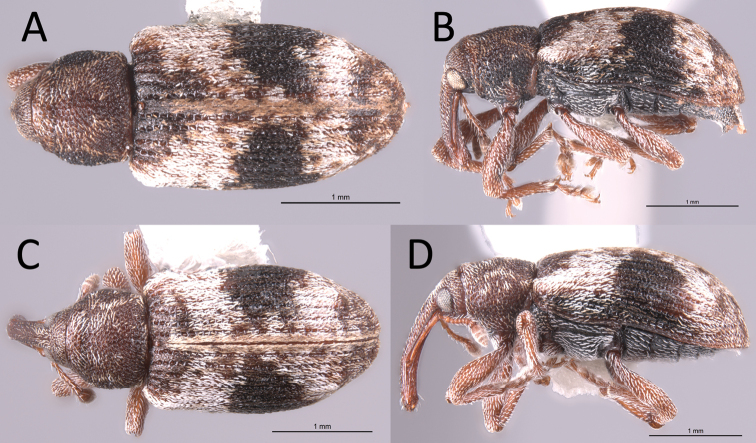
*Proctorusdecipiens* habitus (USI: JHLRSA__PROC_095, JHLRSA_PROC_037) **A** dorsal (♂) **B** lateral (♂) **C** dorsal (♀) **D** lateral (♀).

Figs 2D–F , 3 , 7A, B

Encalus decipiens LeConte, 1876: 213 [type locality: Illinois and Minnesota]. Alonso-Zarazaga and Lyal 1999: 78.
Proctorus decipiens ; LeConte, 1878: 620. O’Brien and Wibmer 1982: 94. Bousquet et al. 2013: 326.

##### Material examined.

***Lectotype* (here designated): USA: Minnesota** (see Notes on types), Type 5349 (1 female, MCZC), MCZ-ENT00005349.

##### Non-type material.

**Canada: Alberta**: Fitzgerald, 14 June 1988, B.F. & J.L. Carr (1, CNCI), JHLRSA_PROC_096; Lethbridge, 16 May 1930, J.H. Pepper (1, CNCI), JHLRSA_PROC_198; Crow’s Nest Pass, 6–7 June 1930, J.H. Pepper (2, CNCI), JHLRSA_PROC_051, JHLRSA_PROC_052; Calgary, 30 June 1957 – 5 July 1958, B.F. & J.L. Carr (4, CNCI), JHLRSA_PROC_191 – JHLRSA_PROC_195; Calgary, 11–12 June 1890, H.C. Fall Collection (1, MCZC), MCZ-ENT00727128; Writing-on-Stone Provincial Park (0.5 miles north), 14 June 1982, R.S. Anderson (3, CMNC), JHLRSA_PROC_041 – JHLRSA_PROC_043; Sturgeon River at Lac Ste. Anne (50°43'N, 114°20'W), 1–3 June 1982, J.S. Richardson (1, CMNC), JHLRSA_PROC_037; Magrath, 20 May 1938, G.S. Walley (1, CNCI), JHLRSA_PROC_140; Medicine Hat, 1 June 1963 – 9 June 1973, B.F. & J.L. Carr (1, CNCI), JHLRSA_PROC_170 – JHLRSA_PROC_173; Edmonton, 8 June 1916 – 1 July 1920, F.S. Carr (2, CNCI; 15 CAS; 1 MCZC; 1 USNM), JHLRSA_PROC_177, JHLRSA_PROC_178, JHLRSA_PROC_210 – JHLRSA_PROC_224, JHLRSA_PROC_337, MCZC00726934; Brule Lake, 29 June 1989, B.F. & J.L. Carr (1, CNCI), JHLRSA_PROC_143; Fort Macleod, 20 June 1976, B.F. & J.L. Carr (2, CNCI), JHLRSA_PROC_101, JHLRSA_PROC_102; Ghost Dam, 1 June 1975, B.F. & J.L. Carr (1, CNCI), JHLRSA_PROC_103; Edson (30 miles west of), 11 June 1950, P. Rubtsoff (1, CAS), JHLRSA_PROC_227; **British Columbia**: Salmon Arm, 9 June 1940, H. Leech (1, MCZC), MCZ-ENT00726945; Brisco, 19 June 1932, O. Bryant (1, CAS), JHLRSA_PROC_241; Terrace, 1927, M.E. Hippisley (2, MCZC), MCZ-ENT0026933; Vancouver, 30 April 1932, G.R. Hopping (4, CAS), JHLRSA_242 – JHLRSA_PROC_245; Vancouver, 24 April 1930, G.H. Larnder (4, RBCM), ENT991-111637, ENT991-111639, ENT991-111640, ENT991-111642; Princeton, Missezula Lake, 16 June 1929, G. Stace Smith, on *Salix* (1, RBCM), ENT991-111638; Harrison, 9 June 1899, A. Hanham (1, RBCM), ENT991112019; Saanich, 12 May 1930, W.H. Preece (1, CNCI), JHLRSA_PROC_067; Creston, 16 June 1950 – 18 April 1956, G. Stace Smith (5, CNCI; 3, CAS), JHLRSA_PROC_109 – JHLRSA_PROC_113, JHLRSA_PROC_246; Revelstoke, 2 June 1978, B.F. & J.L. Carr (1, CNCI), JHLRSA_PROC_196; Fort Steele, 23 May 1977, B.F. & J.L. Carr (3, CNCI), JHLRSA_PROC_188 – JHLRSA_PROC_190; Sicamous, 2 June 1978, B.F. & J.L. Carr (1, CNCI), JHLRSA_PROC_107; Golden (9 miles northwest), 28 August 1973, R.H. Parry (1, CMNC), JHLRSA_PROC_040; Golden, 27 June–30 August 1975, B.F. & J.L. Carr (2, CNCI), JHLRSA_PROC_104, JHLRSA_PROC_105; Robson, June 1949 (1, CNCI), JHLRSA_PROC_135; Vancouver, 30 April 1932, G.R. Hopping (4, CNCI), JHLRSA_PROC_131 – JHLRSA_PROC_134; Lake Errock, near Deroche, 2 June – 4 July 1953, S.D. Hicks, on *Salix* (14, CNCI), JHLRSA_PROC_145 – JHLRSA_PROC_158; Blanket Creek, 9 June 1984, B.F. & J.L. Carr (1, CNCI), JHLRSA_PROC_114; **Manitoba**: Aweme, 24 June 1907 – 27 May 1909, N. Criddle (1, MCZC; 1, USNM), MCZ-ENT00727134, JHLRSA_PROC_336; Russell, 21 July 1954, Brooks-Wallis (1, CNCI), JHLRSA_PROC_050; Winnipeg, 22 May–5 June 1915, J.B. Wallis (1, CNCI; 1, MCZC), JHLRSA_PROC_205, MCZENT-00727131; Riding Mountain Park, 8 June 1937 – 9 June 1938, W.J. Brown (10, CNCI), JHLRSA_PROC_160 – JHLRSA_PROC_169; **New Brunswick**: Boiestown, 13 July 1928, W.J. Brown (1, CNCI), JHLRSA_PROC_059; Carleton County, Wakefield, Meduxnekeag Valley Nature Preserve (46.1931°N, 67.6825°W), 31 May 2005, M.-A. Giguère & R. Webster (1, RWC), JHLRSA_PROC_002; Madawaska County, Gounamitz Road (47.62250°N, 68.96973°W), 21 June 2011, Martin N. Turgeon (1, NBM), NBM-070118; Restigouche County, Summit Area, 7 June 2011, Martin N. Turgeon (1, NBM), NBM-070119; **Northwest Territories**: Fort Simpson, Manners Creek, 11 June 1972, A. Smetana (1, CNCI), JHLRHS_PROC_095; Highway 5 (2 km east of Junction with Highway 2), 16 June 1988, B.F. & J.L. Carr (3, CNCI), JHLRSA_PROC_200 – JHLRSA_PROC_202; Highway 7 (125 km north of British Columbia border), 25 June 1988, B.F. & J.L. Carr (1, CNCI), JHLRSA_PROC_203; Along Highway 7 (219 km north of British Columbia border), 21 June 1988, B.F. & J.L. Carr (1, CNCI), JHLRSA_PROC_204; **Nova Scotia**: Tusket, 27 June 1947, W.J. Brown (1, CNCI), JHLRSA_PROC_141; Hants County, Mount Uniacke, 14 June 1947, W.J. Brown (4, CNCI), JHLRSA_PROC_136 – JHLRSA_PROC_139; Waverley, 10 June 1947, W.J. Brown (1, CNCI), JHLRSA_PROC_144; Bathurst, July 1915, J.N. Knull (1, CAS), JHLRSA_PROC_233; **Ontario**: Toronto, 25 May 1896, R.J. Crew (1, USNM), JHLRSA_PROC_335; Parry Sound, 14 July 1932, G.S. Walley (1, CNCI), JHLRSA_PROC_199; Smoky Falls, Mattagami River, 21 June 1934, G.S. Walley (1, CNCI), JHLRSA_PROC_055; Merivale, 4 May 1937, W.J. Brown (2, CNCI), JHLRSA_PROC_053, JHLRSA_PROC_054; Mer Bleue, Ottawa, 28 May 1935, W.J. Brown (1, CNCI), JHLRSA_PROC_060; Black Rapids, Ottawa, 23 May 1927, W.J. Brown (1, CNCI), JHLRSA_PROC_099; Sultan Road, 6.8 km west of junction with Highway 144, 26 June 1996, B.F. & J.L. Carr (2, CNCI), JHLRSA_PROC_064, JHLRSA_PROC_065; Ottawa, 9 May 1930, W.J. Brown (1, CNCI), JHLRSA_PROC_108; Longlac (13 km west) along Highway 11, 10 June 1995, B.F. & J.L. Carr (1, CNCI), JHLRSA_PROC_206; Pass Lass Junction (13 km southwest), 10 June 1995, B.F. & J.L. Carr (1, CNCI), JHLRSA_PROC_181; Prince Edward County, 1 June 1919 – 23 June 1923, J.F. Brimley (2, CNCI), JHLRSA_PROC_179 – JHLRSA_PROC_180; Rainy River District, 18 June – 7 September 1924, J.F. Brimley (3, CNCI), JHLRSA_PROC_174 – JHLRSA_PROC_176; Hastings County, 14 June 1938 (1, CNCI), JHLRSA_PROC_159; Moosonee, 30 June 1973, J.M. Campbell & Parry (1, CMNC), JHLRSA_PROC_039; Pickle Lake (8 miles north), 19–22 June 1973, J.M. Campbell & Parry (5, CMNC), JHLRSA_PROC_045 – JHLRSA_PROC_049; Lake Superior Provincial Park, Frater, 13 June 1973, J.M. Campbell & R. Parry (1, CMNC), JHLRSA_PROC_035; Lake Superior Provincial Park, Noisy Bay, 13 June 1973, J.M. Campbell & R. Parry (1, CMNC), JHLRSA_PROC_034; Carleton County, Constance Bay, 4 May 1982, H. & A. Howden (1, CMNC), JHLRSA_PROC_036; Thunder Bay District, Stanley, 9–17 June 1981, M. Kaulbars (1, CMNC), JHLRSA_PROC_044; **Quebec**: Aylmer, 16 August 1916, J.N. Knull (1, CAS; 1, MCZC), JHLRSA_PROC_234, MCZ-ENT00727133; Duparquet, 11 June 1936 – 21 June 1944, G. Stace Smith, on *Salix* (5, CAS), JHLRSA_PROC_235 – JHLRSA_PROC_239; Duparquet, 11 June 1936, G. Stace Smith (1, USNM), JHLRSA_PROC_333; Montreal, Liebeck Collection (2, MCZC), MCZ-ENT00726926, MCZ-ENT00726927; Sept-Îles, 8 June 1929, W.J. Brown (1, CNCI), JHLRSA_PROC_061; Temiscamingue County, L’Etang, 16 August 1985, Larochelle & Lariviere (1, CNCI), JHLRSA_PROC_062; Cadillac, 2 July 1981 (1, CNCI), JHLRSA_PROC_063; Knowlton, 14 June 1928, G.H. Fisk (1, CNCI), JHLRSA_PROC_097; Cascapedia, 11–20 June 1938, W.J. Brown (25, CNCI), JHLRSA_PROC_068 – JHLRSA_PROC_092; Forillon National Park, trail off of park compound (48.857°N, 64.376°W), 10–17 June 2013, F. Tremblay (1, CBG), BIOUG11159-H04, JHLRSA_PROC_001; Gatineau, Mont Cascades (45.590941°N, 75.850435°W), 13 May 2021, J.H. Lewis, beaten off *Salix* sp. (1, CMNC); Gatineau, Mont Cascades (45.590941°N, 75.850435°W), 12 May 2021, J.H. Lewis, beaten off *Salix* sp. (1, CMNC); Laurentides Wildlife Reserve (km. 145), 16 June 2012, P. de Tonnancour, beating *Salix* sp. (2, CMNC; 11, PdTC), JHLRSA_PROC_004 – JHLRSA_PROC_016; Laurentides Wildlife Reserve, Mre-du-Sault (km. 117), 16 June 2012, P. de Tonnancour, beating *Salix* sp. (10, PdTC), JHLRSA_PROC_017 – JHLRSA_PROC_026; Sainte-Catherine, 5 June 2007, C. Tessier (1, PdTC), JHLRSA_PROC_027; Mont Rigaud, 20 April 2012, P. de Tonnancour, beating *Salix* sp. (1, PdTC), JHLRSA_PROC_028; Grand-Remous, Chemin Baskatong (46.7729°N, 75.8802°W), 27 May 2017, P. de Tonnancour, beating *Salix* sp. (3, PdTC), JHLRSA_PROC_029 – JHLRSA_PROC_31; Villeroy, 30 May 1990, C. Chantal (1, CCCH), JHLRSA_PROC_032; Quebec, 21 June 1966, C. Chantal (1, CCCH), JHLRSA_PROC_033; **Saskatchewan**: Val Marie, 11 June 1955, A.R. Brooks (1, CNCI), JHLRSA_PROC_100; Elbow, 23 June 1954, Brooks-Wallis (2, CNCI), JHLRSA_PROC_093, JHLRSA_PROC_094; **Yukon**: Eagle River, Dempster Highway, 15 June 1980, R.J. Cannings (1, CMNC), JHLRSA_PROC_038; Kirkman Creek, 13 June 1928 (1, CNCI), JHLRSA_PROC_142; Dempster Highway (mile 123), 2 August 1979, B.F. & J.L. Carr (2, CNCI), JHLRSA_PROC_186, JHLRSA_PROC_187; Dawson, 24–29 June 1924, H.C. Fall Collection (2, MCZC), MCZ-ENT00727135, MCZ-ENT00727136. **USA: Alaska**: Along Wales Highway, Hess Creek (149°10'N, 65°40'W), 10 July 1978, J.M. Campbell & A. Smetana (3, CNCI), JHLRSA_PROC_056 – JHLRSA_PROC_058; Selawik National Wildlife Reserve (66.85873°N, 158.16618°W), 24 June 2010, D.S. Sikes, sweep in open sand dunes (1, UAMIC), UAM100283525; Fairbanks, Chena Ridge (64.79672°N, 148.02143°W), 12 June 2005, D.S. Sikes, silver birch and black spruce forest (4, UAMIC), UAM100361536-UAM100361539; Circle, 21 June 1928 (13, CNCI), JHLRSA_PROC_115 – JHLRSA_PROC_127; Circle Hot Springs, 20 June 1945, J.C. Chamberlin, swept from mustard (1, USNM), JHLRSA_PROC_355; Beaver, 24 June 1928 (3, CNCI; 1, MCZC), JHLRSA_PROC_128 – JHLRSA_PROC_130, MCZ-ENT00727130; **California**: Del Norte County, Smith River Recreational Area (41°47.696'N, 124°02.184'W), 27 June 2002, F.G. Andrews & A.J. Gilbert (1, CMNC), JHLRSA_PROC_003; Trinidad, 7 June 1925, J.O. Martin (1, CAS), JHLRSA_PROC_240; Del Norte County, Gasquet, 21 April 1966, T. Peacock & R.P. Allen, on *Sambucusracemosa* L. (1, USNM), JHLRSA_PROC_347; **Colorado**: Fort Collins, Liebeck Collection (1, MCZC), MCZ-ENT00726929; Garland (3, MCZC Main Collection and Horn Collection), MCZ-ENT00726940, MCZ-ENT00726941, MCZ-ENT00772808; Garland, Hubbard and Schwarz (2, USNM), JHLRSA_PROC_350, JHLRSA_PROC_351; La Veta (1, MCZC (LeConte Collection)), MCZ-ENT00772802; La veta, Hubbard & Schwarz (2, USNM), JHLRSA_PROC_348, JHLRSA_PROC_349; “Colorado”, F.C. Bowditch Collection (1, MCZC), MCZ-ENT00726939; “Col” (2, MCZC (Horn Collection)), MCZ-ENT00772803, MCZ-ENT00772804; **Idaho**: Burley, 2 June 1986, B.F. & J.L. Carr (1, CNCI), JHLRSA_PROC_066; Coolin, Priest Lake, 19 July 1927, E.C. Van Dyke (1, CAS), JHLRSA_PROC_232; Coeurd’ Alene, June, Wickham (2, MCZC), MCZ-ENT00726935, MCZ-ENT00726936; **Maine**: Monmouth, 16 July 1913 – 16 July 1915, C.A. Frost, on *Salix* (2, MCZC), MCZ-ENT00726931, MCZ-ENT00726932; Rockland, July 1893, H.C. Fall Collection (1, MCZC), MCZ-ENT00727129; **Massachusetts**: Stoneham, July 1910, F.A. Sherriff (1, MCZC), MCZ00726943; **Michigan**: Marquette, Hubbard & Schwarz (2, USNM), JHLRSA_PROC_338, JHLRSA_PROC_339; Detroit, Hubbard & Schwarz (1, USNM), JHLRSA_PROC_342; **Minnesota**: Grand Marais, 25 August 1951, Bryant (4, CAS), JHLRSA_PROC_207 – JHLRSA_PROC_209, JHLRSA_PROC_300; Duluth, A. Fenyes Collection (1, CAS), JHLRSA_PROC_231; Ithaca State Park, September 1927, S. Garthside (1, USNM), JHLRSA_PROC_341; **Montana**: Tiber Dam, 28 June 1982, B.F. & J.L. Carr (1, CNCI), JHLRSA_PROC_197; “Mon.” (2, USNM), JHLRSA_PROC_331, JHLRSA_PROC_332; Bear Paw Mountain, Hubbard and Schwarz (2, USNM), JHLRSA_PROC_352, JHLRSA_PROC_353; Bozeman, 31 May 1907 (1, USNM), JHLRSA_PROC_354; **New Hampshire**: Squam Lake, 2 July 1931, J.W. Green (1, CAS), JHLRSA_PROC_301; New York: Cranberry Lake, 20 June 1922, M.H. Hatch (1, USNM), JHLRSA_PROC_340; **Oregon**: Corvallis, June 1919, Liebeck Collection, on willow (1, MCZC), MCZ-ENT00726930; **Utah**: Tony Grove (2 km west) along Highway 89, 24 June 1986, B.F. & J.L. Carr (1, CNCI), JHLRSA_PROC_106; Utah Lake, June 1919, Hubbard and Schwarz (1, CAS), JHLRSA_PROC_302; Utah Lake (2, MCZC (Horn Collection)), MCZ-ENT00772806, MCZ-ENT00772807; Utah Lake, Hubbard & Schwarz (4, USNM), JHLRSA_PROC_342 – JHLRSA_PROC_346; Duchesne, 23 June 1948, G.F. Knowlton (1, USNM), JHLRSA_PROC_334; **Washington**: Olympia, Liebeck Collection (1, MCZC), MCZ-ENT00726928; Monroe, 4–14 July 1906, Van Dyke Collection (3, CAS), JHLRSA_PROC_225, JHLRSA_PROC_226, JHLRSA_PROC_303; Easton, Koebele Collection (2, CAS), JHLRSA_PROC_228, JHLRSA_PROC_229; Everett, July 1912, A. Fenyes Collection (2, CAS), JHLRSA_PROC_230; Everett, July 1912, Wickham (3, MCZC; 5 USNM), MCZ-ENT00726937, MCZ-ENT00726938, MCZ-ENT00727132, JHLRSA_PROC_356 – JHLRSA_PROC_360; Silver Lake, 28 June 1945, Anderson, on willow (5, USNM), JHLRSA_PROC_361 – JHLRSA_PROC_363; Seattle, on willow (1, USNM), JHLRSA_PROC_364; Montesano, 11 August 1944, Forse & Smith, on willow (4, USNM), JHLRSA_PROC_365 – JHLRSA_PROC_368; Yakima County, White Swan, 1 May 1979, B. McAfee (7, USNM), JHLRSA_PROC_369 – JHLRSA_PROC_375; **Wyoming**: Junctions of Highway 120 and 296, 22–27 June 1982, B.F. & J.L. Carr (4, CNCI), JHLRSA_PROC_182 – JHLRSA_PROC_185; “Wy” (1, MCZC (Horn Collection)), MCZ-ENT00772805; **UNSPECIFIED LOCALITY**: Central United States (1, MCZC (LeConte Collection)), MCZ-ENT00772800.

##### Notes on types.

This species was described based on two specimens collected from Illinois and Minnesota. One syntype specimen is deposited in the MCZC (see specimens examined) and is likely the Minnesota syntype as it bears a circular blue label that LeConte used to indicate specimens collected around Lake Superior. The specimen bears six labels: a circular blue label, a square brown label reading “2008”, a square red type label reading “Type. 5349”, a rectangular brown ID label reading “P.decipiens Lec. (Encalus)”, a rectangular white label reading “Jan.–Jul. 2005 MCZ Image Database”, and a rectangular white label reading “MCZ-ENT00005349”. The location of the second syntype is unknown. Here, we designate the examined *P.decipiens* syntype (MCZ-ENT00005349) as a lectotype to fix the identity of this species.

**Figure 4.**
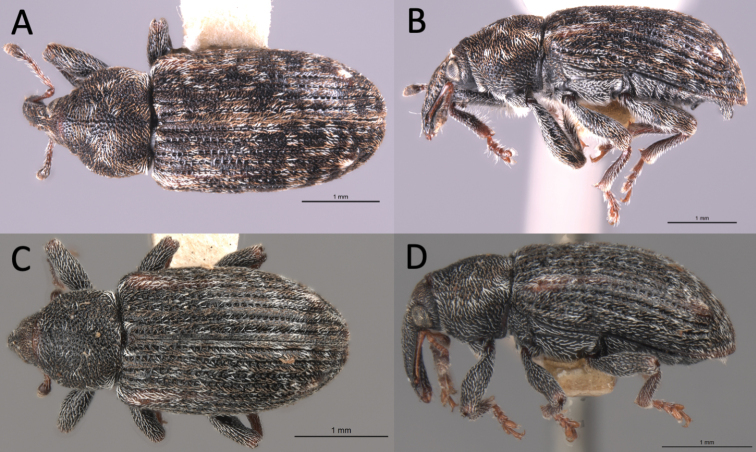
*Proctorusarmatus* habitus (USI: JHLRSA_PROC_318, MCZ-ENT00005224) **A** dorsal (♂) **B** lateral (♂) **C** dorsal (♀) **D** lateral (♀).

##### Diagnosis.

Length 2.9–3.1 mm. Body (especially rostrum and femora) rufous. Protibiae of male not prominently dentate on inner edge. Elytra with clear, distinct x-pattern of white scales. Fifth ventrite of male with two prominent ventral apico-lateral projections which are connected by a transverse ridge; without any baso-medial ventral projection. Apical tooth of metatibiae of male curved. Aedeagus with apex with margins weakly expanded laterally, and coming to weak, rounded point.

##### Ecology.

This species has been collected frequently from *Salix* and is occasionally taken in large numbers. The record of one specimen (JHLRSA_PROC_347) collected from *Sambucusracemosa* L. is likely incidental and does not reflect use of *Sambucus* as a host.

#### ﻿ Proctorus truncatus

Taxon classificationAnimaliaColeopteraCurculionidae

Lewis & Anderson sp. nov.

13269936-B6C5-570C-945E-8423552047D0

https://zoobank.org/172594D2-4FA6-4AEB-B275-91D0172173AD

**Figure 5.**
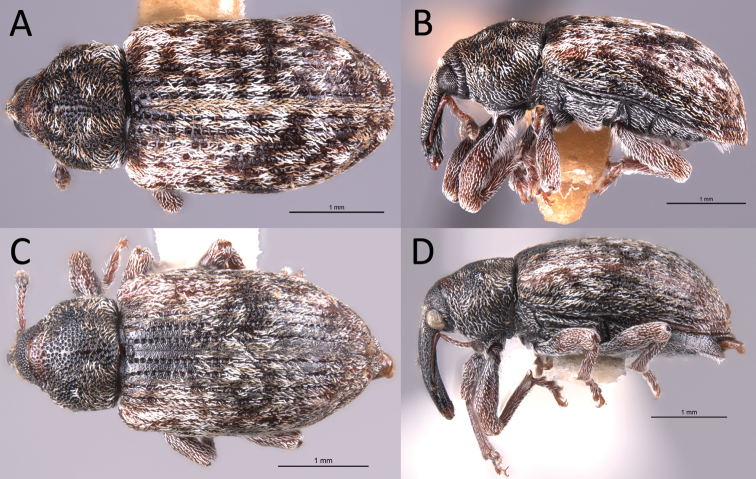
*Proctorustruncatus* habitus (USI: JHLRSA_PROC_305, JHLRSA_PROC_282) **A** dorsal (♂) **B** lateral (♂) **C** dorsal (♀) **D** lateral (♀).

Figs 1A–C , 5 , 7E, F , 8B

##### Material examined.

***Holotype*: Canada: Ontario**: Constance Bay, 17 May 2003, H. & A. Howden (1 male, CMNC), JHLRSA_PROC_292. ***Paratypes*: Canada: Alberta**: Ghost Dam, 25 May 1983, B.F. & J.L. Carr (1, CNCI), JHLRSA_PROC_299; Tp. 12, Rge. 1, 11 April 1976, B.F. & J.L. Carr (1, CNCI), JHLRSA_PROC_304; Tp. 34, Rge. 4, 21 May 1962, B.F. & J.L. Carr (1, CNCI), JHLRSA_PROC_311; Olds, T.N. Willing (1, USNM), JHLRSA_PROC_328; **British Columbia**: Paul Lake, 24 May 1933, A. Thrupp (3, CAS), JHLRSA_PROC_269 – JHLRSA_PROC_271; Terrace, M.E. Hippisley (1, MCZC), MCZ-ENT00726924; **Manitoba**: Aweme, 5 May 1898 – 12 May 1914, N. Criddle, on *Populus* (1, CNCI; 2 MCZC), JHLRSA_PROC_306, MCZ-ENT00726919, MCZ-ENT00726920; **New Brunswick**: Northumberland County, Parker, 3 June 1959, on Aspen (1, AFCF), AFCF0019835; York County, Durham, 5 May 1958, G.W. Barter, on *Populustremuloides* Michx. (1, AFCF), AFCF0019834; Fredericton, 29 April 1913 (1, CNCI), JHLRSA_PROC_298; **Ontario**: Constance Bay, 17 May 2003, H. & A. Howden (1, CMNC), JHLRSA_PROC_293; Honey Harbor, 10 June 1932, G.S. Walley (1, CNCI), JHLRSA_PROC_305; Lake Superior Provincial Park, Old Woman Bay, 11 June 1973, M. Campbell and R. Parry (1, CMNC), JHLRSA_PROC_295; Lake Superior Provincial Park, Sand River, 6 June 1973, M. Campbell and R. Parry (1, CMNC), JHLRSA_PROC_294; Bell’s Corner, 2 June 1950, S.D. Hicks (1, CNCI), JHLRSA_PROC_297; Rainy River District, 18–20 June 1924, J.F. Brimley (1, CNCI; 1 MCZC), JHLRSA_PROC_309, MCZ-ENT00726925; Rainy River, 11 June 1924, J.F. Brimley (1, USNM), JHLRSA_PROC_326; Sudbury, 1889 (1, CNCI), JHLRSA_PROC_310; **Quebec**: Harrington Lake, 1 June 1954, H.J. Huckel (1, CNCI), JHLRSA_PROC_308; Duparquet, 11 June 1935–26 May 1944, G. Stace Smith, on *Populustremuloides* Michx. (18, CAS; 2, CMNC; 1, USNM), JHLRSA_PROC_247 – JHLRSA_PROC_266, JHLRSA_PROC_329; St. Rose, 1 June 1939, G. Stace Smith, on *Pinuscontorta* Douglas ex Loudon (1, CAS), JHLRSA_PROC_267; Saint Hyppolyte, Biology station of Laurentides (45.9778°N, 74.0039°W), 3 June 2017, P. de Tonnancour, beaten from *Populusgrandidentata* Michx. (2, CMNC; 8, PdTC), JHLRSA_PROC_272 – JHLRSA_PROC_281; Saint-Pierre, 3 June 1984, C. Chantal, from *Populusgrandidentata* Michx. (2, CMNC; 8, CCCH), JHLRSA_PROC_282 – JHLRSA_PROC_291; **Saskatchewan**: Cut Knife, 29 August 1940, A.R. Brooks (1, CNCI), JHLRSA_PROC_307; Montreal River, 26 May 1954, on *Populus* (1, CNCI), JHLRSA_PROC_296; **USA: Michigan**: Marquette (1, MCZ (Horn Collection))), MCZ-ENT00772799; **Minnesota**: “Min.”, H.C. Fall Collection (1, MCZC), MCZ-ENT00726922; **New Hampshire**: Mount Washington (summit), 29 July 1954, Becker, Munroe & Mason (1, CNCI), JHLRSA_PROC_312; **New Mexico**: Santa Fe, 14 June 1935, Van Dyke Collection (1, CAS), JHLRSA_PROC_268; **New York**: Chateaugay Lake, F.C. Bowditch (1, MCZC), MCZ-ENT00726921; **Utah**: Logan, 29 April 1934, T.O. Thatcher (1, USNM), JHLRSA_PROC_327.

##### Diagnosis.

Length 3.7–4.0 mm. Body (especially rostrum and femora) dark, although elytra often has orange stripe extending posteriorly from humerus. Protibiae of male not prominently dentate on inner edge. Elytra without clear, distinct x-pattern of white scales. Fifth ventrite of male with two low, minutely serrate ridges apico-laterally, which are connected by a transverse ridge; fifth ventrite also with a single, smaller ventral projection positioned baso-medially. Apical tooth of metatibiae of male straight. Aedeagus with apex dorsoventrally flattened and expanded laterally.

##### Etymology.

The specific name refers to the truncate ventral projections on the fifth ventrite of males (compare with *P.armatus* which has long ventral projections).

##### Ecology.

This species has been collected from *Populusgrandidentata* Michx. and *P.tremuloides* Michx. The single record of a specimen (JHLRSA_PROC_267) from *Pinuscontorta* Douglas ex Loudon is likely incidental.

##### Taxonomic comments.

This species was long confused with the less common *P.armatus* from which it differs in the armature of the fifth ventrite (males), protibia dentation (males), genitalia (males), rostrum length (females) and overall body shape (both sexes). LeConte (1878: 620) wrote of *P.armatus* (two years after describing that species): “Several specimens of this curious insect were found at Marquette, and among them are ♂♂ in which the two processes of the apical edge of the fifth ventral segment are very short, and scarcely apparent, though the anterior tubercle or spine and the large excavation are as well developed as in the other specimens.” Clearly, LeConte is referring to our new species *P.truncatus*; however, he apparently assumed that the differences in ventrite armature were cases of intraspecific variation in *P.armatus* as no additional species were described.

#### ﻿ Proctorus emarginatus

Taxon classificationAnimaliaColeopteraCurculionidae

Lewis & Anderson sp. nov.

4884B236-6E55-5FF7-99A1-FF681ED7E593

https://zoobank.org/4BD64D73-88C9-46ED-A0CE-C8FDE063FA01

**Figure 6.**
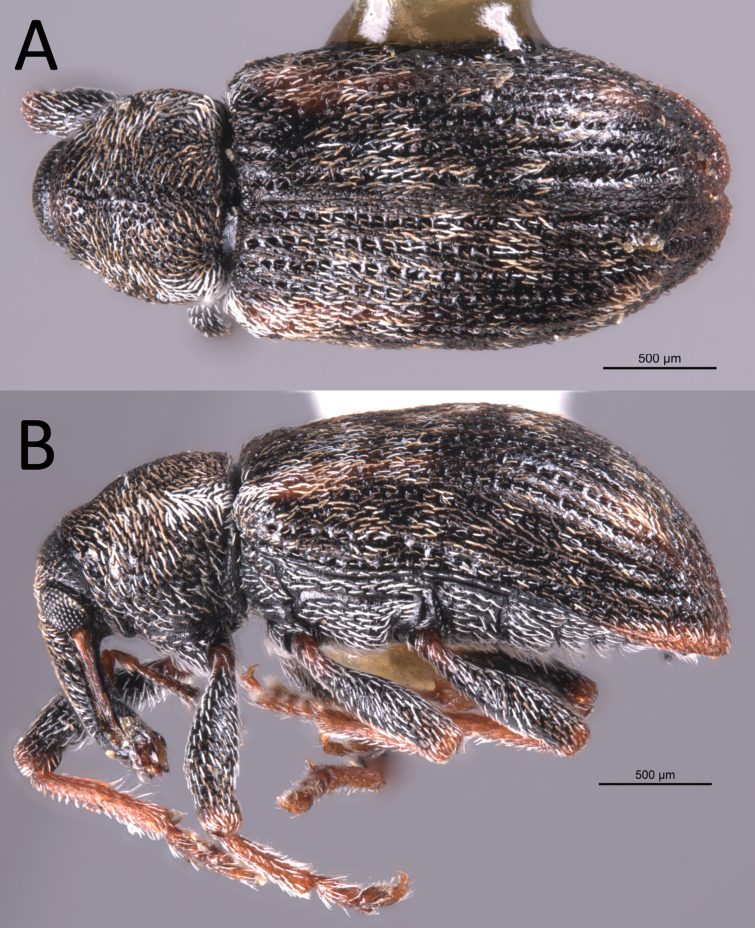
*Proctorusemarginatus* habitus (USI: JHLRSA_PROC_325) **A** dorsal (♂) **B** lateral (♂).

**Figure 7.**
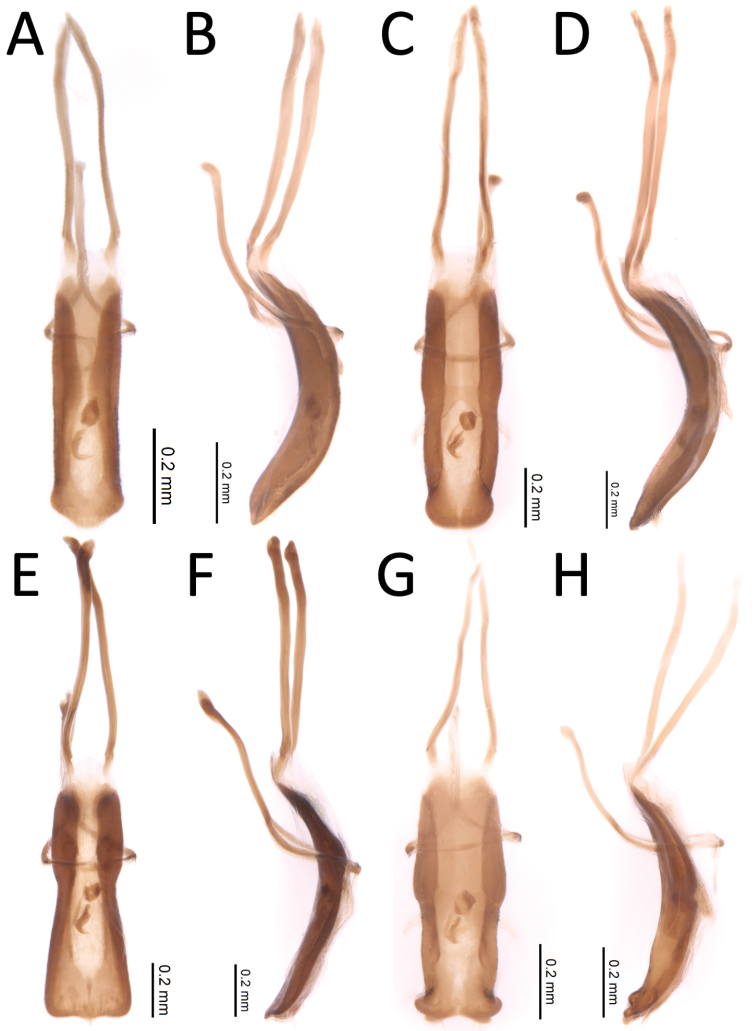
Aedeagi of four *Proctorus* species. *Proctorusdecipiens* (USI: JHLRSA_PROC_035) **A** dorsal **B** lateral. *Proctorusarmatus* (USI: JHLRSA_PROC_316) **C** dorsal **D** lateral; *Proctorustruncatus* (USI: JHLRSA_PROC_295) **E** dorsal **F** lateral; *Proctorusemarginatus* (USI: JHLRSA_PROC_325) **G** dorsal **H** lateral.

Figs 2A–C , 6 , 7G, H

##### Material examined.

***Holotype*: Canada: British Columbia**: Summit Lake (Alaska Highway – mi. 392), 25 June 1959, R.E. Leech, on *Salix* (1 male, CNCI), JHLRSA_PROC_325. ***Paratypes*: Canada: Alberta**: Tp. 78, Rge. 15, 5 June 1984, B.F. & J.L. Carr (1, CMNC), JHLRSA_PROC_323; **Northwest Territories**: Highway 5 (49 km, east of junction with Highway 2), 16 June 1988, B.F. & J.L. Carr (1, CNCI), JHLRSA_PROC_324.

##### Diagnosis.

Length 2.9–3.1 mm. Body (especially rostrum and femora) dark, although elytra often has orange stripe extending posteriorly from humerus. Protibiae of male not prominently dentate on inner edge. Elytra without clear, distinct x-pattern of white scales. Fifth ventrite of male with a single transverse ridge which peaks medially; without any baso-medial ventral projection. Apical tooth of metatibiae of male straight. Aedeagus with apex distinctly emarginate and with four prominent lobes (two on each side).

##### Etymology.

The specific name refers to the apically emarginate body of the penis.

##### Ecology.

One specimen was collected from *Salix*. However, nothing else is known of the natural history of this species.

##### Remarks.

This species is known only from northwestern North America (only Canada at present), and based on institutional collection records also represents one of the rarer weevils in Canada. The female of *P.emarginatus* is unknown.

**Figure 8.**
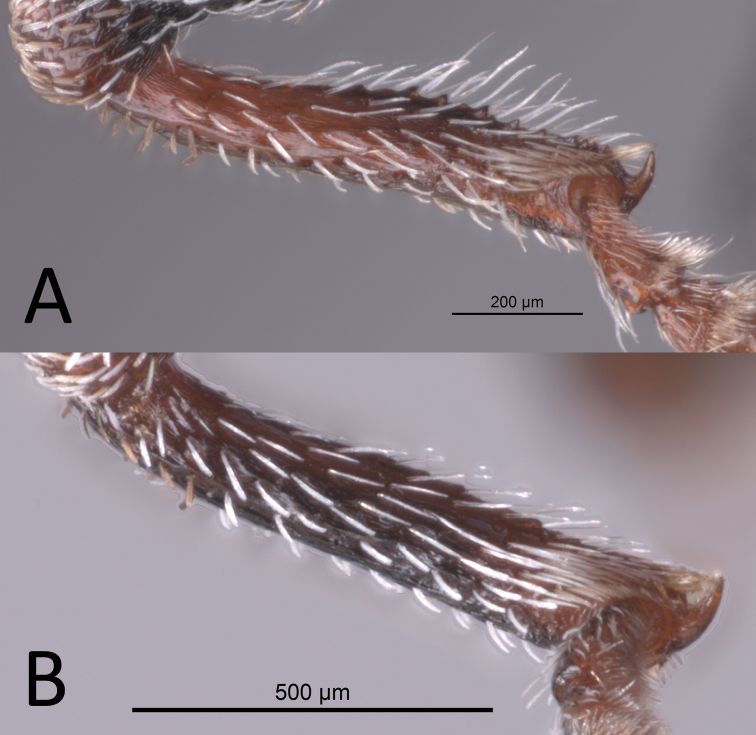
Male protibiae **A***Proctorusarmatus***B***Proctorustruncatus*.

### ﻿Key to the species of *Proctorus* LeConte

Note that the female of *P.emarginatus* is unknown and therefore not included in the key.

**Table:** 

1	Fifth ventrite with armature (projections, swellings) (Figs 1, 2, 3B, 4B, 5B, 6B) [males]	**2**
–	Fifth ventrite unmodified (Figs 3D, 4D, 5D) [females]	**5**
2	Fifth ventrite lacking basomedial swelling, but with two apicolateral projections (Figs 2D–F, 3B). Apical tooth of metatibiae curved. Femora and usually rostrum reddish (Fig. 3A, B). Elytra with distinctive x-shaped pattern of white scales (Fig. 3A, B). Apex of penis with edges weakly expanded laterally, and coming to weak, rounded point (Fig. 7A, B)	***Proctorusdecipiens* (LeConte, 1876) (male)**
–	Fifth ventrite never with combination of apicolateral projections and lack of basomedial swelling (Figs 1, 2A–C, 4B, 5B, 6B). Apical tooth of metatibiae straight. Femora and usually rostrum dark (Figs 4A, B, 5A, B, 6A, B). Elytra without discernible pattern of scales (Fig. 4A, B, 5A, B, 6A, B). Penis not as above (Fig. 7C–H)	**3**
3	Fifth ventrite with two prominent apicolateral projections and basomedial swelling (Fig. 1D–F, 4B). Apical half of protibiae strongly dentate on ventral side (Fig. 8A). Apex of penis slightly rounded to truncate, not emarginate or significantly expanded laterally (Fig. 7C, D)	***Proctorusarmatus* LeConte, 1876 (male)**
–	Fifth ventrite not as above, always lacking two prominent apicolateral projections (Figs 1A–C, 2A–C, 5B, 6B). Apical half of protibiae not dentate or only with weak dentation ventrally (Fig. 8B). Apex of penis dorsoventrally flattened and expanded laterally (Fig. 7E, F) or emarginate (Fig. 7G, H)	**4**
4	Fifth ventrite with prominent basomedial swelling and low, apicolateral ridges (Figs 1A–C, 5B). Apex of penis dorsoventrally flattened and expanded laterally (Fig. 7E, F). Length 3.7–4.0 mm	***Proctorustruncatus* sp. nov. (male)**
–	Fifth ventrite lacking prominent basomedial swelling, but with apical ridge that swells to a peak medially (Figs 2A–C, 6B). Apex of penis distinctly emarginate and with four prominent lobes (two on each side) (Fig. 7G, H). Length 2.9–3.1 mm	***Proctorusemarginatus* sp. nov. (male)**
5	Cuticle reddish, with distinctive x-shaped pattern of white scales across elytra (Fig. 3C, D)	***Proctorusdecipiens* (LeConte, 1876) (female)**
–	Cuticle dark, without discernible pattern of scales on elytra (Figs 4C, D, 5C, D)	**6**
6	Body more round in dorsal and lateral view (Fig. 5C, D). Rostrum thinner and longer (Fig. 5D)	***Proctorustruncatus* sp. nov. (female)**
–	Body more elongate and somewhat flattened dorsoventrally (Fig. 4C, D). Rostrum thicker and shorter (Fig. 4D)	***Proctorusarmatus* LeConte, 1876 (female)**

## ﻿Discussion

*Proctorus* represents a morphologically distinct monophyletic lineage that is endemic to North America. The specific armature on the fifth ventrite of males is unique to the genus within North America; however, we note that the males of the Old World species *Dorytomusdorsalis* (Linnaeus, 1758) also possesses similar structures. Although *Proctorus* is currently placed in the subtribe Ellescina with *Ellescus* (Alonso-Zarazaga and Lyal 1999; Anderson 2002; Bousquet et al. 2013; Caldara et al. 2014; Alonso-Zarazaga et al. 2017), the shared ventral armature in *Proctorus* and *Dorytomus* (subtribe Dorytomina) hints at a closer phylogenetic relationship between those genera than previously thought. Preliminary molecular work suggests that *Proctorus* represents a lineage sister to or nested within *Dorytomus*. However, improved taxon and gene sampling is required to improve branch support and determine which of those phylogenetic hypotheses is correct (unpublished data, Lewis and Anderson). Here, we take a conservative approach and continue to recognize the validity of *Proctorus* as members of that genus are morphologically separable from all *Dorytomus* species (including *D.dorsalis*) by tarsal claw morphology.

Although the species of *Proctorus* are easily distinguished by external and internal morphology, two species described here were long overlooked. This is likely due to the fact that specimens of the genus are rare in institutional collections. Indeed, *P.emarginatus* sp. nov. is only known from three specimens. Future studies of the genus should focus on surveying for the female of *P.emarginatus* and further investigating differing host plant usage amongst the species.

## Supplementary Material

XML Treatment for Proctorus

XML Treatment for Proctorus armatus

XML Treatment for Proctorus decipiens

XML Treatment for Proctorus truncatus

XML Treatment for Proctorus emarginatus
